# Comparison of vitamin D, parathyroid hormone, and body composition in obese premenopausal and postmenopausal women

**DOI:** 10.1007/s12020-026-04652-1

**Published:** 2026-05-04

**Authors:** Burcu Çaykara Peran, Hilal Adil, Güler Öztürk, Mehmet Sargın, Hacer Hicran Mutlu

**Affiliations:** 1 Department of Medical Biology, Hamidiye Faculty of Medicine, University of Health Sciences, Istanbul, Türkiye Türkiye; 2 Department of Physiology, Faculty of Medicine, Usak University, Usak, Türkiye; 3 Department of Physiology, Faculty of Medicine, Istanbul Medeniyet University, Istanbul, Türkiye Türkiye; 4 Department of Family Medicine, Faculty of Medicine, Istanbul Medeniyet University, Istanbul, Türkiye Türkiye

**Keywords:** Obesity, Vitamin D, Premenopause, Postmenopause

## Abstract

**Purpose:**

We aimed to investigate the associations of serum vitamin D and parathyroid hormone (PTH) with body composition parameters, including bone weight and mineral content, in obese premenopausal and postmenopausal women.

**Methods:**

In this retrospective study, 200 pre-menopausal and 200 post-menopausal obese women were evaluated. Body composition was assessed using a bioelectrical impedance analyzer (TANITA-48M), and biochemical parameters were measured by standard laboratory methods.

**Results:**

The mean serum vitamin D level was significantly lower in pre-menopausal women compared with post-menopausal women (16.91 ± 13.17 vs. 22.59 ± 16.49 ng/mL, p < 0.05). Overall, 83.75% (n = 335) of the study population had vitamin D deficiency or insufficiency, while only 16.25% (n = 65) demonstrated normal vitamin D levels. BMI did not differ significantly between the two groups (p > 0.05). In pre-menopausal women, serum vitamin D levels were negatively correlated with PTH and mineral content. In post-menopausal women, vitamin D levels showed a negative correlation with BMI, body weight, body fat percentage, and PTH (p < 0.05).

**Conclusions:**

The prevalence of hypovitaminosis D was remarkably high (83.75%) among obese women. Moreover, declining vitamin D levels were associated with increased body weight and adiposity, as well as elevated PTH.

## Background

Overweight and obesity affect approximately one-third of the global population and are associated with an increased risk of chronic diseases such as diabetes mellitus and cardiovascular disease [1]. Body mass index (BMI) is widely used for the classification of overweight (25.0–29.9 kg/m²) and obesity (≥ 30.0 kg/m²), with obesity further categorized into class I (30.0–34.9 kg/m²), class II (35.0–39.9 kg/m²), and class III (≥ 40.0 kg/m²) [2]. Obesity is characterized by chronic low-grade inflammation and metabolic disturbances in adipose tissue, which become more pronounced in older individuals. Post-menopausal women, in particular, exhibit increased visceral adiposity compared with younger women, who are relatively protected from adipose tissue inflammation [3].

Vitamin D deficiency has been consistently linked with both general [4] and abdominal obesity [5]. Serum vitamin D concentrations are inversely associated with body fat percentage and waist-to-height ratio, particularly in women [6]. Experimental studies demonstrated that high-fat diets reduce hepatic 25-hydroxylase (CYP2R1) mRNA expression, which correlates with lower circulating vitamin D levels [7]. In addition, sequestration of vitamin D within adipose tissue has been proposed to reduce its bioavailability [8].

Vitamin D is primarily synthesized in the skin upon ultraviolet exposure, with a minor contribution from dietary intake [9]. 7-Dehydrocholesterol (7-DHC), which is formed by exposure to sunlight, is transported to the liver by circulation. In the liver, 7-DHC is converted to 25-hydroxyvitamin D [25(OH)D_3_], the major circulating form, and subsequently to the active metabolite 1,25-dihydroxyvitamin D [1,25(OH)₂D] in the kidney via 1α-hydroxylase (CYP27B1) [10]. Beyond its classical role in calcium-phosphate homeostasis, 1,25(OH)₂D regulates numerous biological processes including cell proliferation, differentiation, apoptosis, and DNA repair via interaction with the vitamin D receptor [11]. The parathyroid hormone (PTH) is a major regulator of renal 1,25(OH)₂D synthesis [12], while 1,25(OH)₂D itself exerts negative feedback on PTH gene transcription and modulates calcium-phosphate balance in concert with fibroblast growth factor 23 (FGF23) [13].

Although vitamin D deficiency has been linked to obesity and altered PTH regulation, it remains unclear whether these associations differ according to menopausal status in obese women. This question is clinically relevant because menopause is accompanied by major hormonal and metabolic changes that may affect adiposity and calcium–vitamin D homeostasis. Therefore, the primary objective of this study was to compare serum 25(OH)D and PTH levels, together with body composition parameters, between obese premenopausal and postmenopausal women. By examining these relationships in the same clinical cohort, our study provides additional evidence on the interplay between obesity, menopausal status, and vitamin D–PTH balance.

## Materials and methods

### Study Design and Participants

This retrospective, cross-sectional study was conducted using medical records of obese women who attended the Obesity Outpatient Clinic of the Department of Family Medicine, Istanbul Medeniyet University Göztepe Training and Research Hospital (IMU-GTRH). Women aged 25–60 years who had available measurements of serum vitamin D and parathyroid hormone (PTH) were included. Body composition was assessed using the TANITA-48 M bioelectrical impedance analyzer in routine outpatient practice. The device-generated outputs included body weight, fat mass, fat percentage, fat-free mass, muscle mass, basal metabolic rate, and BIA-derived estimates of bone weight and mineral content. In addition to body composition measurements, parameters such as “bone weight” and “mineral content” were obtained using the TANITA-48 M bioelectrical impedance analysis (BIA) device in routine clinical practice. BIA provides indirect estimates for “bone weight” and “mineral content,” and these parameters were interpreted as approximate indicators of body composition.

Only the initial test results obtained at the first clinical visit were included in the analysis. Medical records of 427 obese women (body mass index ≥ 30 kg/m²) aged 25–65 years were initially screened. Records with incomplete biochemical or body composition data were excluded, resulting in a final study population of 400 obese women, including 200 premenopausal and 200 postmenopausal participants. Menopausal status was determined based on clinical records. Women with regular menstrual cycles were classified as premenopausal, whereas women with documented natural menopause (absence of menstruation for at least 12 consecutive months) were classified as postmenopausal.

Serum 25(OH)D₃, PTH, calcium, and phosphorus concentrations were measured as part of routine clinical evaluation in the hospital’s biochemistry laboratory. Serum calcium and phosphorus levels were determined using routine automated clinical chemistry analyzers, whereas serum 25(OH)D₃ and PTH concentrations were measured using automated immunoassay-based methods under standardized laboratory conditions. The same laboratory protocols were applied to all participants throughout the study period.

Due to the retrospective nature of the study, detailed analytical specifications, including assay manufacturers, detection limits, and inter-assay coefficients of variation, were not available from archival records. Nevertheless, all biochemical measurements were performed in the same institutional laboratory, ensuring methodological consistency across the study population.

## Definitions

Serum vitamin D levels were classified as follows: deficiency, < 20 ng/mL; insufficiency, 20–29 ng/mL; sufficiency, ≥ 30 ng/mL; and intoxication, > 150 ng/mL. The laboratory reference ranges were as follows: PTH, 15–65 pg/mL; serum calcium, 8.5–10.3 mg/dL; and phosphorus, 3–4.5 mg/dL.

### Statistical Analysis

All statistical analyses were performed using SPSS version 22.0 (IBM Corp., Armonk, NY, USA). Data distribution was assessed with the Shapiro–Wilk test. Group comparisons were carried out using Student’s t-test or Mann–Whitney U test, as appropriate. Correlations were examined using Pearson or Spearman’s rho correlation coefficients. To evaluate whether menopausal status modified the association between adiposity and serum vitamin D levels, multiple linear regression analyses were performed. *p* < 0.05 was considered statistically significant.

## Results

**Fig. 1:**
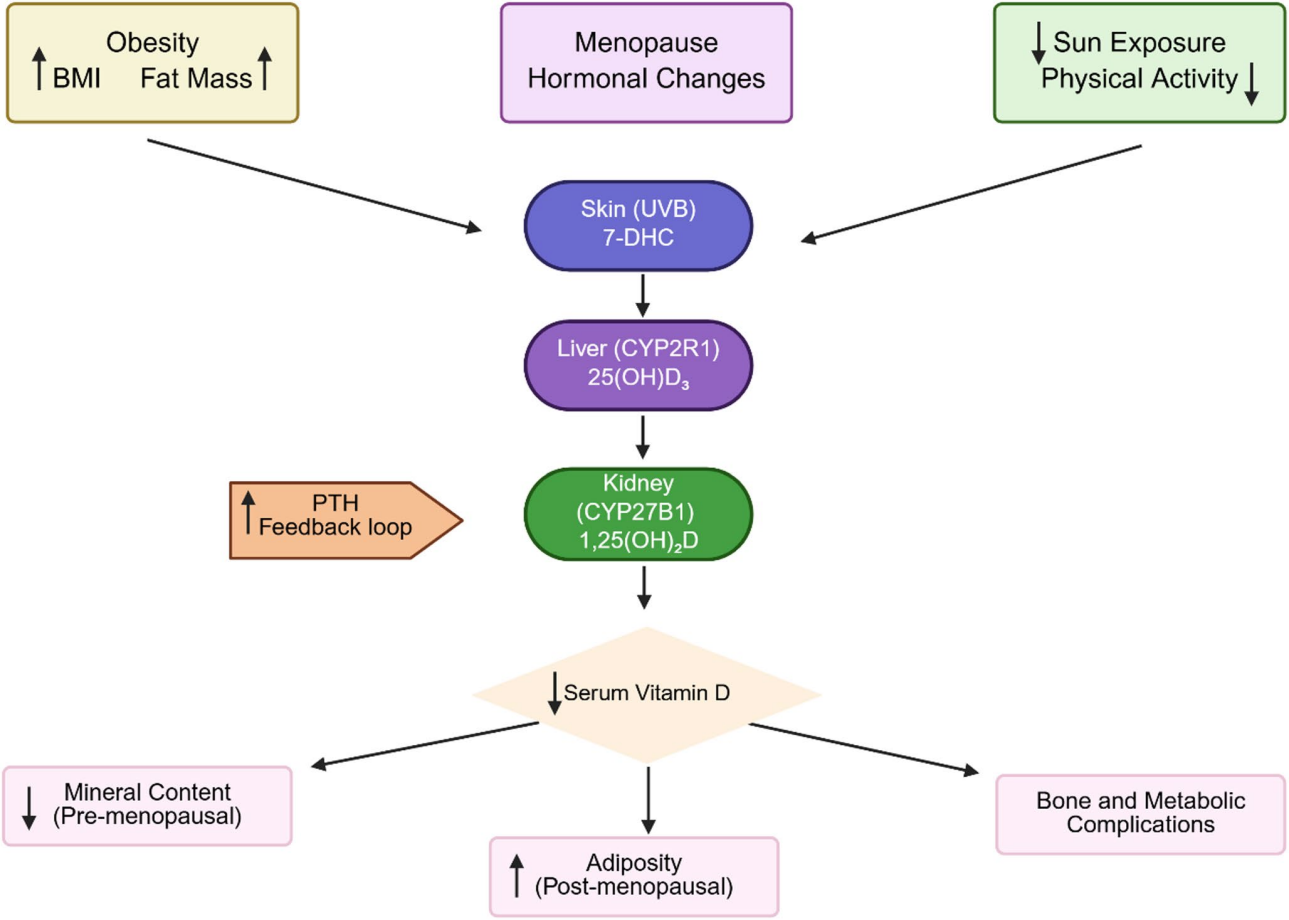
Conceptual model linking obesity, menopause, vitamin D metabolism, and bone health outcomes

The mean age of the study population was 47.0 ± 11.4 years. When stratified by menopausal status, the mean ages were 38.4 ± 9.0 years in the pre-menopausal group and 55.7 ± 5.4 years in the post-menopausal group. Mean height was 160.4 ± 5.7 cm in pre-menopausal women and 156.8 ± 5.7 cm in post-menopausal women, with an overall mean of 158.6 ± 6.0 cm. Mean body weight was 97.3 ± 16.0 kg in pre-menopausal and 93.5 ± 14.1 kg in post-menopausal women, with an overall mean of 95.4 ± 15.2 kg. Baseline anthropometric, biochemical, and body composition parameters (BMI, serum 25(OH)D₃, PTH, calcium, phosphorus, bone weight, fat mass and percentage, basal metabolic rate, fat-free mass, mineral and protein content) are presented in Table 1. Significant differences were observed between pre- and post-menopausal women for age, weight, height, 25(OH)D₃, calcium, phosphorus, bone weight, muscle mass, fat-free mass, mineral content, and basal metabolic rate (*p* < 0.05). Figure 1 illustrates the conceptual model of our findings.

Obesity (increased BMI and fat mass), menopausal hormonal changes, and reduced sun exposure or physical activity are major risk factors contributing to decreased serum vitamin D concentrations. Vitamin D metabolism involves cutaneous synthesis of 7-DHC upon UVB exposure, hepatic hydroxylation to 25(OH)D₃ via CYP2R1, and renal conversion to the biologically active metabolite 1,25(OH)₂D through CYP27B1. Reduced serum vitamin D levels are associated with upregulated PTH levels and distinct outcomes depending on menopausal status: decreased mineral content in pre-menopausal women and increased adiposity in post-menopausal women. Collectively, these alterations contribute to bone and metabolic complications in obese women (It needs to be written like this: (Figure 1 is created in BioRender. Çaykara Peran, B. (2026) https://BioRender.com/hva2log).

**Table 1:** Average parameter values of pre and post-menopausal obese women

Parameters	Premenopausal			Postmenopausal			*P* value	Total		
	Mean ± SD	Min	Max	Mean ± SD	Min	Max		Mean ± SD	Min	Max
**Age (year)**	38.4 ± 9	20	60	55.7 ± 5.4	39	65	**< 0.001**	47 ± 11.4	20	65
**Height (cm)**	160.4 ± 5.7	144	178	156.8 ± 5.7	142	172	**< 0.001**	158.6 ± 6	142	178
**Weight (kg)**	97.3 ± 16	70.4	158	93.5 ± 14.1	66.9	139.8	**0.019**	95.4 ± 15.2	66.9	158
**BMI (kg/m** ^**2**^ **)**	37.8 ± 5.7	30	56.7	38 ± 5.5	30.1	56.3	0.481	37.9 ± 5.6	30	56.7
**25OHD**_**3**_ **(ng/dL)**	16.9 ± 13.2	2.2	84.8	22.6 ± 16.5	3.4	136	**< 0.001**	19.8 ± 15.2	2.2	136
**PTH (pg/mL)**	48 ± 22.7	9.2	144	47.2 ± 35.8	3.9	418	0.113	47.6 ± 30	3.9	418
**Ca (mg/dL)**	9.4 ± 0.4	8.3	10.6	9.6 ± 0.4	8.5	11.5	**< 0.001**	9.5 ± 0.4	8.3	11.5
**P (mg/dL)**	3.5 ± 0.5	2.3	5.1	3.7 ± 0.5	2.6	6.2	**< 0.001**	3.6 ± 0.5	2.3	6.2
**Bone weight (kg)**	2.9 ± 0.3	2.1	3.9	2.8 ± 0.3	2.1	4	**< 0.001**	2.9 ± 0.3	2.1	4
**Fat (kg)**	39.2 ± 10.7	21.9	84.8	38 ± 9	19	67.2	0.444	38.6 ± 9.9	19	84.8
**Fat (%)**	39.7 ± 4.5	28.3	53.8	40.3 ± 4.3	30	52.4	0.145	40 ± 4.4	28.3	53.8
**Muscle (kg)**	55.2 ± 6	39.3	74.4	52.6 ± 6.3	38.8	76.5	**< 0.001**	53.9 ± 6.3	38.8	76.5
**BMR (kcal)**	1791.9 ± 212.3	1294	2505	1689.8 ± 210.6	1247	2491	**< 0.001**	1740.9 ± 217.3	1247	2505
**FFM (kg)**	58.1 ± 6.3	41.4	78.3	55.4 ± 6.6	40.9	80.5	**< 0.001**	56.8 ± 6.6	40.9	80.5
**Mineral amount (kg)**	4.2 ± 0.5	2.7	5.7	3.5 ± 0.5	2.3	5.9	**< 0.001**	3.8 ± 0.6	2.3	5.9
**Protein amount (kg)**	12.4 ± 1.4	8.9	16.6	12.5 ± 1.5	9	17.5	0.318	12.4 ± 1.4	8.9	17.5

PTH; Parathyroid hormone, Ca; Calcium, P; Phosphorus, BMI; Body mass index, BMR; Basal metabolic rate, FFM; Free fat mass, SD; Standard deviation, Min; Minimum, Max; Maximum

According to obesity classification, 71 pre-menopausal and 68 post-menopausal women were categorized as class I obese, 77 and 61 as class II obese, and 52 and 71 as class III obese, respectively (Table 2).

**Table 2:** Grouping of pre and post-menopausal obese women according to body mass index levels

Premenopausal					Postmenopausal					*P* value
BMI	Number	Mean ± SD	Min	Max	BMI	Number	Mean ± SD	Min	Max	*P*
**Total**	200 (100%)	37.8 ± 5.735	30	56.7	**Total**	200 (100%)	38.01 ± 5.49	30.1	56.3	0.481
**30–34.99 kg/m** ^**2**^	71 (35.5%)	32.61 ± 1.58	30	34.9	**30–34.99 kg/m** ^**2**^	68(34%)	32.38 ± 1.46	30.1	34.9	0.357
**35–39.99 kg/m** ^**2**^	77(38.5%)	37.27 ± 1.47	35	39.9	**35–39.99 kg/m** ^**2**^	61(30.5%)	37.27 ± 1.53	35	39.9	0.869
**> 39.99 kg/m** ^**2**^	52(26%)	45.66 ± 4.57	40	56.7	**> 39.99 kg/m** ^**2**^	71(35.5%)	44.03 ± 3.68	40	56.3	0.054

BMI; Body mass index, SD; Standard deviation, Min; Minimum, Max; Maximum

The mean serum 25(OH)D₃ level was significantly lower in pre-menopausal women compared with post-menopausal women (16.91 ± 13.17 vs. 22.59 ± 16.49 ng/mL, *p* < 0.001). Overall, 83.75% of participants (*n* = 335) had vitamin D deficiency or insufficiency, while only 16.25% (*n* = 65) demonstrated normal vitamin D levels (Table 3).

**Table 3:** Grouping of pre and post-menopausal obese women by vitamin D levels

Premenopausal					Postmenopausal					*P* value
Vitamin D (25OHD_3_) Levels	Number	Mean ± SD	Min	Max	Vitamin D (25OHD_3_) Levels	Number	Mean ± SD	Min	Max	*P*
**Total**	200 (100%)	16.91 ± 13.17	2.2	84.8	**Total**	200 (100%)	22.59 ± 16.49	3.4	136	< 0.001
**0–10 ng/dL**	72 (36%)	6.87 ± 1.9	2.2	10	**0–10 ng/dL**	38 (19%)	7.4 ± 1.944	3.4	10	0.153
**10–20 ng/dL**	77 (39%)	14.6 ± 3.07	10.1	19.7	**10–20 ng/dL**	61 (31%)	15.22 ± 3.17	10.2	20	0.222
**20–30 ng/dL**	27 (14%)	24.71 ± 2.9	20.4	28.9	**20–30 ng/dL**	60 (30%)	24.58 ± 3.24	20.1	30	0.773
**30–150 ng/dL**	24 (12%)	45.65 ± 13.52	31	84.8	**30–150 ng/dL**	41 (21%)	44.72 ± 22.58	30.4	136	0.101

SD; Standard deviation, Min; Minimum, Max; Maximum

Correlation analyses revealed a negative association between serum 25(OH)D₃ and both PTH and mineral content in pre-menopausal women. In post-menopausal women, 25(OH)D₃ levels were inversely correlated with BMI, body weight, body fat, and PTH levels (*p* < 0.05; data not shown).

Multiple linear regression analysis was performed to evaluate the independent predictors of serum vitamin D levels. The overall model was statistically significant but demonstrated limited explanatory power (F = 8.88, *p* < 0.001; *R* = 0.287; R² = 0.083; adjusted R² = 0.073). Age was identified as the only independent predictor of vitamin D levels (B = 0.369, SE = 0.10, β = 0.276, *p* < 0.001). BMI, menopausal BMI parameter, and sample type were not significantly associated with vitamin D levels (*p* > 0.05) (data were not shown).

## Discussion

The mean serum 25(OH)D₃ level was significantly lower in pre-menopausal women compared with post-menopausal women (16.91 ± 13.17 vs. 22.59 ± 16.49 ng/mL, *p* < 0.05). Only 16.25% of participants exhibited sufficient vitamin D concentrations, while the majority demonstrated deficiency or insufficiency. Correlation analyses revealed an inverse relationship between serum 25(OH)D₃ and BMI, body weight, fat mass, and PTH levels in post-menopausal women (*p* < 0.05). In pre-menopausal women, serum 25(OH)D₃ was inversely correlated with PTH and mineral content (*p* < 0.05). However, although the regression model was statistically significant, its explanatory power was low. This finding suggested that a significant portion of the variation in vitamin D levels was determined by factors outside the variables included in the model. The fact that vitamin D is influenced by numerous environmental and behavioral determinants such as sunlight exposure, seasonal variability, dietary intake, supplement use, and lifestyle factors supports this finding.

Vitamin D deficiency, defined as serum 25(OH)D₃ <30 nmol/L, is particularly prevalent among adolescents, pregnant women, and the elderly [14]. The Endocrine Society (2011) defines vitamin D deficiency as < 20 ng/mL and recommends serum levels ≥ 30 ng/mL to ensure optimal musculoskeletal health [15]. The primary cause of vitamin D deficiency is inadequate sunlight exposure, although other contributing factors include low dietary intake of vitamin D, seasonal variation, and socioeconomic conditions. Endogenously, previtamin D₃ is synthesized from 7-DHC in the skin under ultraviolet B radiation (290–315 nm), followed by hepatic conversion to 25(OH)D₃ and renal hydroxylation to the biologically active metabolite 1,25(OH)₂D₃ [16].

The prevalence of vitamin D deficiency is markedly elevated in individuals with obesity, which represents a major global health problem. Several mechanisms have been proposed to explain this association. One is the volumetric dilution hypothesis, whereby vitamin D is distributed across a larger pool of fat, serum, liver, and muscle tissue, resulting in lower circulating concentrations [17]. Another factor may be reduced sun exposure due to lower physical activity levels, limiting cutaneous synthesis of vitamin D [18]. Population-based studies consistently report a high prevalence of hypovitaminosis D in obese women. For instance, vitamin D deficiency (serum 25(OH)D₃ <50 nmol/L) was observed in approximately 80% of Saudi women, with multivariate analyses identifying BMI, sun exposure index, vitamin D supplementation, waist-to-hip ratio, and age as independent predictors [19]. In a Turkish cohort, vitamin D deficiency was present in 37% and insufficiency in 50% of obese participants, with a stronger association in women and a negative correlation between serum 25(OH)D₃ and BMI [20]. Consistent with these findings, our study demonstrated that 83.75% of obese women had either vitamin D deficiency or insufficiency, while only 16.25% exhibited sufficient levels. In our study, parameters associated with adiposity (BMI and menopausal-BMI) were not found to be independently associated with vitamin D levels in this cohort. While there are studies in the literature reporting a relationship between adiposity and vitamin D levels, the lack of an association in the current analysis can be explained by cohort-specific characteristics and residual confounding factors.

Previous studies have reported conflicting findings regarding the relationship between BMI, menopausal status, and vitamin D levels. Biglia et al. observed that higher BMI was positively correlated with larger tumor size in both pre- and post-menopausal women; however, they did not classify participants by obesity categories [21]. In contrast, Chikwati et al. reported no significant differences in BMI between pre- and post-menopausal women [22]. In line with these observations, our study also demonstrated similar BMI values in both groups (*p* > 0.05), and further stratification according to obesity class revealed no significant differences (*p* > 0.05).

With respect to vitamin D, one study including obese men and women found no significant differences in serum concentrations between obese and non-obese groups. Nonetheless, when vitamin D sufficiency status was considered, a significant association emerged between obesity and vitamin D deficiency [23]. Similarly, in our cohort, post-menopausal women had higher mean serum 25(OH)D₃ concentrations than pre-menopausal women (22.59 ± 16.49 vs. 16.91 ± 13.17 ng/mL, *p* < 0.001). However, when participants were categorized based on sufficiency cut-off levels, no significant differences were observed between groups (*p* > 0.05). Kim et al. reported mean serum 25(OH)D₃ levels of 17.16 ± 6.28 ng/mL in pre-menopausal and 20.20 ± 7.69 ng/mL in post-menopausal women, and further demonstrated a direct association between vitamin D concentrations and abdominal obesity in post-menopausal women [24]. Our findings are consistent with these results, indicating higher vitamin D levels in obese post-menopausal women compared to their pre-menopausal counterparts. In our study, only age, not BMI or menopausal status, was identified as the sole independent predictor of vitamin D levels. This may be related to differences in health behaviors, vitamin supplement use, or access to healthcare among age groups. However, more comprehensive datasets are needed to clearly elucidate the underlying biological or behavioral mechanisms of this relationship.

Berridge et al. reported that vitamin D deficiency may contribute to earlier onset of menopause [25]. Furthermore, supplementation with vitamin D and calcium has been shown to exert beneficial effects on bone mineral density, supporting their use as a preventive strategy against early postmenopausal bone loss [26]. Masaon et al. demonstrated that weight loss achieved through low-calorie diets or exercise leads to increased circulating 25(OH)D₃ levels in post-menopausal women [27]. In addition, calcium supplementation has been associated with reduced fat accumulation and increased fat-free mass in the trunk region of post-menopausal women, even in the absence of BMI changes [28]. Recent clinical data support the idea that vitamin D and calcium supplementation may have positive effects on bone health indicators [29]. In our study, muscle mass was significantly higher in pre-menopausal women compared to post-menopausal women, which was reflected in higher basal metabolic rate (BMR) and fat-free mass (FFM) values. Moreover, mineral content was also significantly greater in pre-menopausal women (*p* < 0.001). These findings are consistent with the known effects of menopause-related hormonal changes, particularly estrogen deficiency, which leads to reductions in lean mass, BMR, and bone mineral parameters in post-menopausal women.

An inverse relationship between serum 25(OH)D₃ and fat mass in post-menopausal women has been previously reported [30]. The combined evaluation of vitamin D and PTH is reported to have clinical significance, particularly in terms of diagnostic and treatment strategies for endocrine-related bone metabolism disorders [31]. Our findings demonstrated a negative correlation between 25(OH)D₃, PTH, and mineral content in pre-menopausal women, while in post-menopausal women, 25(OH)D₃ was inversely correlated with BMI, body weight, fat mass, and PTH levels (*p* < 0.05). In another study comparing obese pre-menopausal and overweight post-menopausal women, serum calcium was significantly lower and PTH significantly higher in the post-menopausal group [32]. In our study, calcium, phosphorus, and bone mass differed significantly between pre- and post-menopausal women (*p* < 0.001). Interestingly, PTH levels did not differ significantly between the two groups (*p* > 0.05).

Our results are in line with previous studies showing that hypovitaminosis D is highly prevalent in obese women and that obesity is an independent risk factor for reduced circulating vitamin D levels. However, few studies to date have simultaneously addressed the interaction between obesity, vitamin D, PTH, and body composition across menopausal stages. Although correlation patterns differed between pre- and postmenopausal women, multivariable analyses did not support an independent modifying effect of menopausal status. Future studies that include variables such as lifestyle variables, seasonal factors, and vitamin supplement use in the model may improve the performance of models predicting vitamin D levels.

Several limitations have been identified in our study. First, the retrospective and cross-sectional design precludes any causal inference. Second, potential confounding factors such as sunlight exposure, dietary intake, physical activity, seasonal variation, and vitamin D supplementation were not controlled, which may have influenced the observed associations. Third, the study was conducted in a single center, thereby limiting the generalizability of the findings and although BIA is a non-invasive and practical tool for estimating overall body composition, its “bone weight” and “mineral content” outputs cannot be equated with bone mineral content obtained from dual-energy X-ray absorptiometry (DEXA) [33]. DEXA is the accepted gold standard for measuring bone mineral content and density, and the absence of DEXA measurements in our study prevents us from drawing definitive conclusions regarding skeletal bone health. Fourth, although multivariable analyses were performed, residual confounding cannot be excluded due to the observational nature of the study.

Despite these limitations, the study provides novel insights into the high prevalence of vitamin D deficiency among obese women and underscores the urgent need for preventive and therapeutic strategies in populations at increased risk for bone and metabolic complications associated with obesity and menopause.

## Conclusion

In this cohort of 400 obese women, the prevalence of vitamin D deficiency or insufficiency (< 30 ng/mL) was remarkably high at 83.75%. Lower vitamin D concentrations were associated with higher body weight and PTH levels. Our results suggest that supplementation strategies to correct low vitamin D levels in obese women, regardless of menopausal status, may be considered.

## Data Availability

The datasets generated and analyzed during the current study are available from the corresponding author on reasonable request.

## Abbreviations

1,25(OH)₂D: 1,25-dihydroxyvitamin D
7-DHC: 7-Dehydrocholesterol
25(OH)D_3_: 25-hydroxyvitamin D
BMI: Body mass index
CYP27B1: 1α-hydroxylase
CYP2R1: 25-hydroxylase
FGF23: Fibroblast growth factor 23
IMU-GTRH: Istanbul Medeniyet University Göztepe Training and Research Hospital
PTH: Parathyroid hormone
